# Policy options to deal with high-cost medicines – survey with European policy-makers

**DOI:** 10.1186/2052-3211-8-S1-P8

**Published:** 2015-10-05

**Authors:** Nina Zimmermann, Sabine Vogler, Hanne Bak Pedersen

**Affiliations:** 1 WHO Collaborating Centre for Pharmaceutical Pricing and Reimbursement Policies, Health Economics Department, Gesundheit Österreich GmbH (Austrian Public Health Institute), Vienna, 1010, Austria; 2 Health Technologies and Pharmaceuticals, Division of Health Systems and Public Health, WHO Regional Office for Europe, Copenhagen, 2100, Denmark

## Background

The affordability and financing of new, frequently high-cost, medicines pose challenges to governments world-wide. In Europe, the continual introduction of new premium-priced medicines is of special concern and requires adapted policy options. The aim of the study was to survey whether and which pricing and reimbursement policy options European countries have implemented for new premium-priced medicines.

## Methods

A cross-country survey was carried out through the instrument of a Pharmaceutical Pricing and Reimbursement Information (PPRI) query with policy-makers responsible for pharmaceutical pricing and reimbursement in 42 countries, thereof all 28 European Union (EU) Member States and nine further European countries. Responses were received from 26 European countries and Canada between February and March 2014.

## Results

Most respondents reported that they had no specific definition for high-cost and/or premium-priced medicines in their country, although they were clearly aware of the issue. Most countries responded that they did not yet have specific policies for the pricing and reimbursement of premium-priced medicines versus other medicines. Several countries reported about the use of managed-entry agreements, such as risk-sharing schemes and discount/rebate arrangements. In addition, the relevance of Health Technology Assessments (HTA) and pharmaco-economic evaluations for these medicines was highlighted by some countries. Countries were working on in-patient policies in particular since high-cost and premium-priced medicines are often used in the hospital setting. Some European countries implemented specific funding models: While medicines used in the in-patient sector are usually funded out of the hospital budget (DRG funding), specific high-cost and/or premium- priced medicines are financed on an individual product basis (e.g. Belgium, Finland). Another option is funding of such medicines out of special funds (the Cancer Drugs Funds in England). Horizon scanning was reported from a few countries (Canada, Italy, the UK).

## Conclusions

Although European governments were concerned with the cost issue due to new medicines, specific pricing and reimbursement policies have yet to be thought through in a systematic manner. Prioritization processes will increasingly be required for the introduction of new medicines. They should incorporate the principles of collaboration and transparency: Cooperation between countries in Europe and stakeholder dialogues could be further strengthened. This needs to involve better balancing of the value of innovation with equitable, affordable patient access.

